# Vitamin D Receptor Gene Polymorphisms and Breast Cancer Risk in Kazakhstan

**DOI:** 10.5195/cajgh.2013.95

**Published:** 2014-01-24

**Authors:** Ainur Akilzhanova, Zhannur Abilova, Nurgul Sikhayeva, Ivan Shtefanov, Abay Makishev, Tasbolat Adylkhanov, Tolebay Rakhypbekov, Zhaxybay Zhumadilov, Kuvat Momynaliev

**Affiliations:** 1 Center for Life Sciences, Nazarbayev University, Astana, Kazakhstan; 2 National Center for Biotechnology, Almaty, Kazakhstan; 3 Astana Medical University, Astana, Kazakhstan; 4 Semey State Medical University, Semey, Kazakhstan

**Keywords:** breast cancer, gene polymorphysm, vitamin D receptors, genetype suseptibility

## Abstract

**Introduction:**

The steroid hormone 1,25-dihydroxyvitamin D3 is thought to protect against breast cancer. The activity of 1,25-dihydroxyvitamin D3 is mediated via the vitamin D receptor (VDR), and a number of polymorphisms in the *VDR* gene have been identified. These result in distinct genotypes, some of which may alter susceptibility to breast cancer. Two common single nucleotide polymorphisms (SNP) in the *VDR* gene (*VDR*), rs1544410 (BsmI) and rs2228570 (FokI), have been inconsistently associated with breast cancer risk. Increased risk has been reported for the FokI ff genotype, which encodes a less transcriptionally active isoform of *VDR*. A reduced risk has been reported for the BsmI BB genotype which may influence VDR mRNA stability.

**Aim:**

We have investigated whether specific *VDR* gene polymorphisms are associated with breast cancer risk in Kazakhstan women.

**Material and Methods:**

In a case–control study, female breast cancer patients (*315*) and a female control group (*n=604*) were tested for two *VDR* polymorphisms. Statistical analysis was conducted using SPSS19.0.

**Results:**

*:* The *VDR* rs2228570 (FokI) polymorphism was associated with an increased occurence of BC [rs2228570 (folk) ff vs. FF genotype: OR=1.71; 95% CI=1.21–2.43]. No association was noted between rs1544410 (BsmI) BB and breast cancer risk [OR=0.68; 95% CI=0.49–0.95].

**Conclusion:**

*:* Although the factors that increase breast cancer susceptibility remain uncertain, future large studies should integrate genetic variation in VDR with biomarkers of vitamin D status. Additional testing on the effect of varying genotypes on the functional mechanisms of the *VDR* could help to improve future testing and treatment of woman at risk for breast cancer.

